# Student assistantships in orthopedic surgery: A strategy to counteract the declining interest in surgical careers

**DOI:** 10.1007/s00132-025-04645-4

**Published:** 2025-03-26

**Authors:** Yasmin Youssef, Suzanne Zeidler, Christoph-Eckhard Heyde, Tobias Schöbel

**Affiliations:** Department of Orthopedics, Trauma and Plastic Surgery, University of Leipzig, Liebigstraße 20, 04103 Leipzig, Germany

**Keywords:** Surgical education, Staff recruitment, Career choices, Medical student, Practical training, Chirurgische Ausbildung, Personaleinstellung, Berufswahl, Medizinstudent, Praktische Ausbildung

## Abstract

**Introduction:**

The field of orthopedics and trauma surgery in Germany is facing an increasing shortage of young specialists. Several solutions have already been proposed to attract new residents. This study evaluates the influence of a student job in an orthopedic surgery department on the career choice and skill acquisition of medical students.

**Methods:**

A single center, cross-sectional survey was conducted among 51 medical students employed as paid student assistants in the Department of Orthopedics, Trauma and Plastic Surgery at the University of Leipzig since 2017. A total of 42 participants (response rate: 82.4%) completed an online questionnaire assessing sociodemographic data, career choices and skills acquired during their employment. Statistical analysis was performed using SPSS.

**Results:**

Half of the participants with completed medical studies chose surgical residencies, with 83.3% of these in orthopedic surgery. The experience as a student assistant was ranked as the second most influential factor for career decisions after the final practical year. Participants reported significant skill acquisition, particularly in practical ward activities (mean Likert scale: 4.40 ± 0.79), operating room skills 3.55 ± 1.14 and understanding clinical processes 3.45 ± 0.86; however, limited impact was observed on scientific motivation (2.29 ± 1.13) and conducting orthopedic-specific examinations (1.98 ± 0.79).

**Conclusion:**

Student assistantships in orthopedic surgery provide substantial practical training and positively influence career decisions, potentially countering the declining interest in surgical fields. These roles represent an effective strategy for junior staff recruitment, offering structured exposure to the surgical profession. Future research should explore broader multicenter implementations to confirm these findings.

**Graphic abstract:**

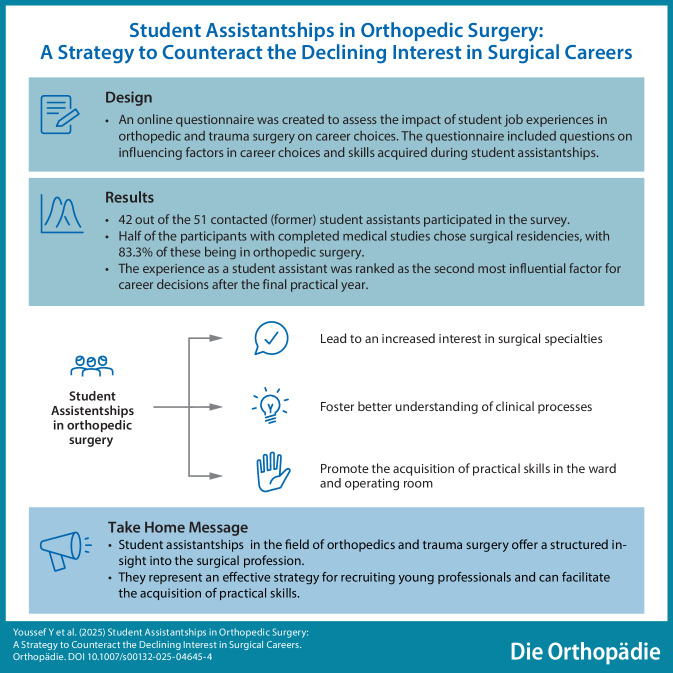

**Supplementary Information:**

The online version of this article (10.1007/s00132-025-04645-4) contains an additional questionnaire, which is available to authorized users.

## Abstract (de)

**Hintergrund:**

Der Fachbereich der Orthopädie und Unfallchirurgie in Deutschland sieht sich mit einem zunehmenden Mangel an jungen Fachkräften konfrontiert. Es wurden bereits mehrere Lösungsansätze zur Gewinnung neuen Nachwuchses vorgeschlagen. Diese Studie evaluiert den Einfluss eines Studentenjobs in einer orthopädisch-chirurgischen Abteilung auf die Berufswahl und den Erwerb von Fähigkeiten von Medizinstudenten.

**Methoden:**

Es wurde eine monozentrische Querschnittsbefragung unter 51 Medizinstudierenden durchgeführt, die seit 2017 als bezahlte studentische Hilfskräfte in der Klinik für Orthopädie, Unfall- und Plastische Chirurgie der Universität Leipzig tätig sind. Insgesamt 42 Teilnehmer (Rücklaufquote: 82,4 %) füllten einen Online-Fragebogen aus, der soziodemografische Daten, Berufswahl und während der Beschäftigung erworbene Fähigkeiten erfasste. Die statistische Analyse wurde mit SPSS durchgeführt.

**Ergebnisse:**

Die Hälfte der Teilnehmer mit abgeschlossenem Medizinstudium entschied sich für eine chirurgische Facharztausbildung, davon 83,3 % in der Orthopädie und Unfallchirurgie. Die Erfahrung als studentische Hilfskraft wurde als zweitwichtigster Einflussfaktor für die Berufsentscheidung nach dem letzten praktischen Jahr genannt. Die Teilnehmer berichteten über einen bedeutenden Erwerb von Fertigkeiten, insbesondere bei praktischen Tätigkeiten auf der Station (mittlere Likert-Skala: 4,40 ± 0,79), Fertigkeiten im Operationssaal (3,55 ± 1,14) und Verständnis für klinische Prozesse (3,45 ± 0,86). Begrenzte Auswirkungen wurden hingegen bei der wissenschaftlichen Motivation (2,29 ± 1,13) und der Durchführung orthopädiespezifischer Untersuchungen (1,98 ± 0,79) beobachtet.

**Schlussfolgerung:**

Studentenjobs im Bereich der Orthopädie und Unfallchirurgie bieten eine umfangreiche praktische Ausbildung und beeinflussen die Berufsentscheidung positiv, was dem sinkenden Interesse an chirurgischen Fächern entgegenwirken könnte. Sie stellen eine wirksame Strategie zur Rekrutierung von Nachwuchskräften dar und bieten einen strukturierten Einblick in den chirurgischen Beruf. Zukünftige Forschungen sollten breitere, multizentrische Implementierungen untersuchen, um diese Ergebnisse zu bestätigen.

## Introduction

In Germany it has been shown that the tendency of medical students to start a surgical residency sinks during medical school from 21% in the first to 13% in the ninth semester [15]. It is expected that Germany is going to face a perceptible lack of young trauma and orthopedic surgeons. It is therefore of great importance to counteract this shortage by recruiting new young talent, already during the medical studies [9]. Previous studies have presented different approaches to achieve this. For example, intensified and practical curricular courses and teaching [4, 10, 16], extracurricular activities, events and grants [5, 11, 17] and student curricula [5, 8]. In addition, it has been proposed that student jobs in trauma and orthopedic surgery departments as surgery and ward assistants can have a positive effect on the motivation of students to start a career in the field [13].

Since August 2017, the Department of Orthopedics, Trauma and Plastic Surgery of the University of Leipzig has offered medical students from the third year of studies the opportunity to work as student assistant. The students’ tasks included assisting in the operating room, taking blood samples and inserting peripheral venous catheters, providing support on the wards, in the central emergency room and the surgical outpatient clinics.

The following study tries to show what influence the experience during student jobs has on career choices of medical students who work in the department of orthopedic and trauma surgery as surgery and ward assistants.

## Methods

### Study design

An online questionnaire was created to assess what influence the experience during student jobs has on career choices of medical students who work in the department of orthopedic and trauma surgery of our institution. LimeSurvey (LimeSurvey GmbH, Hamburg, Germany) was used as online questionnaire software. The link to the questionnaire was sent to all past and current medical students who were working as student assistants in the department. The study was conducted in October 2024. Participation in the online questionnaire was voluntary and anonymity was guaranteed. No identifying data, except for age and gender were collected.

### Questionnaire

The questionnaire was developed based on a review of the current literature on the topic of talent recruitment in surgical fields. The questionnaire was pretested among three independent colleagues, who were involved in the supervision of student assistants.

The final questionnaire included 33 questions and was separated into 3 sections. The first section included questions on sociodemographic data, the current level of training and personal career planning. The second section included questions on what factors influence students choice for a medical field. The third section included questions on what skills were acquired while working as student assistants (Supplement 1).

### Participants

The possibility to assist as student assistant in the operating room and the surgical ward has been offered to medical students since August 2017. The prerequisite was the completion of the first German state examination in medicine, so that only students in the clinical study section (semester 5 upwards) were employed. In total, 53 students worked as student assistants until October 2024, of whom 44 had already left their job at the time of the survey and 7 students were still employed as student assistants at the time of the survey. Of the students two who were currently employed were excluded because they had only been working at the clinic for less than 6 months. Therefore, 51 participants could be included.

### Data processing and statistical analysis

Excel (Microsoft, Seattle, WA, USA) and SPSS (SPSS® for Mac, version 26.0, Chicago, IL, USA) were used for statistical analysis and graphical presentation of the data. Only complete questionnaires were included in the final data analysis. Data were compared using the Mann-Whitney U‑test or Fishers’ exact t‑test. Significance was set at *p* < 0.05.

## Results

### Sociodemographic data

Of the 51 (former) student assistants contacted, 42 (82.4%) completed the questionnaire. Approximately two thirds of the respondents were female (61.9%). The average age was 27.3 years (SD ± 2.9 years). At the time of the survey 18 of the participants (42.9%) were still students, while 24 participants (57.1%) had already completed their studies.

**Fig. 1:**
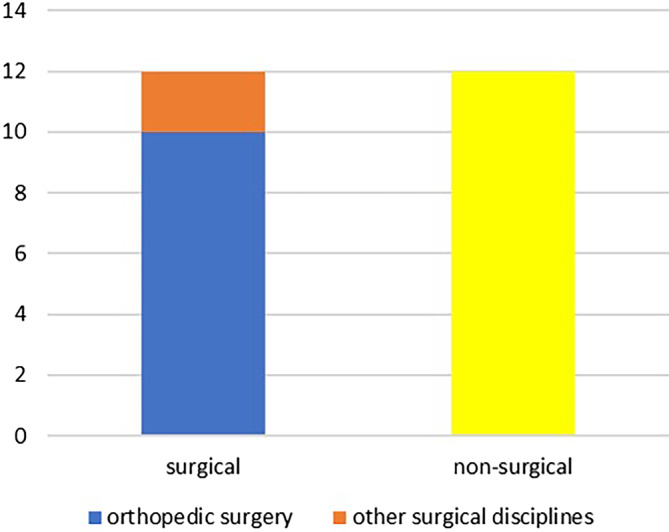
Current professional fields of participants who have completed their medical studies (number of participants)

**Fig. 2:**
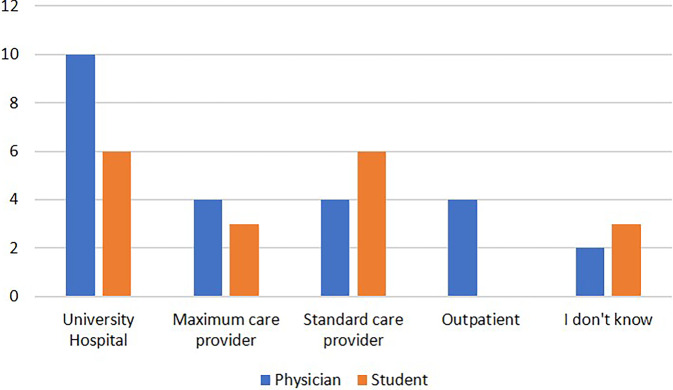
Current workplaces of practicing physicians and career aspirations of participating students (number of participants)

Only one of the participants (2.4%) had already completed specialist training. When asked about their current medical field, 50% of the participants with completed state examination in medicine stated to work in surgery and 50% stated to work in a nonsurgical medical field. Most of the surgical residents (83.3%) worked in orthopedic surgery (Fig. 1). None of the absolvents stated to work nonclinical (e.g. in the medicine industry or primarily as educator). There was no statistically significant influence of gender and the participants decision between a surgical or nonsurgical field (*p* = 0.37) and the decision for or against a career in orthopedic surgery (*p* = 0.39). Most of the participating physicians (41.7%) worked at a university hospital, only four (16.7%) worked in the outpatient sector. The workplace of the participating physicians as well as the career planning (“where do you want to work after completion of the medical state examination?”) of the participating students is shown in Fig. 2. Almost all participating physicians (91.7%) wanted to complete their specialist training in the field they had started. Only two physicians (residents in orthopedic surgery) stated that they wanted to switch from specialist training to general medicine.

**Fig. 3:**
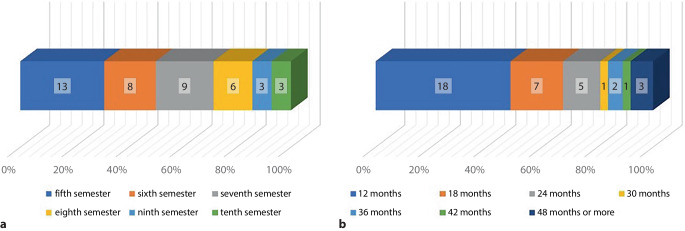
Starting semester and duration of (former) students working as student assistants

On average, the students began their employment between the 6th and 7th semesters (Fig. 3a) and worked as student assistants for an average of 18.6 months (SD: ± 8.8 months). Most of the student assistants worked for 12 months (42.9%), with some participants (7.1%) working over 4 years at the hospital (Fig. 3b). Most participants (88.1%) stated to have been interested in orthopedic surgery before they started the job as student assistant. There was no statistically significant difference between the interest in orthopedic surgery and the participants decision between a surgical or nonsurgical field (*p* = 0.6) or the decision for or against a career in orthopedic surgery (*p* = 0.6). The participants showed a tendency of the student assistant job to influence their chosen field of specialization. There was a tendency of the participants stating that working as a student assistant has influenced the choice of the current or desired field of specialization (mean Likert scale value: 3.62).

### Acquired skills

The answers to what skills were acquired while working as surgery and ward assistants and statements on career and further training on a Likert scale from 1 (completely disagree) to 5 (completely agree) are shown in Table 1.

**Table 1:** Acquired skills and influence of the activity as student assistant on career and further training

Statements on the influence of the activity as student assistant on career and further training: Likert scale from 1–5 (1 = completely disagree to 5 = completely agree)	Mean value and standard deviation
Working as a student assistant helped me to learn practical activities on the ward (blood sampling, peripheral intravenous catheters, etc.)	4.40 (SD: ±0.79)
Working as a student assistant has helped me to build and expand my network	3.21 (SD: ±1.18)
Working as an student assistant helped me to learn practical skills in the operating room	3.55 (SD: ±1.14)
Working as a student assistant has helped me to carry out clinical orthopedic/traumatological examinations	1.98 (SD: ±0.79)
Working as a student assistant has helped me to learn skills at the level of clinical practical communication and organization	3.40 (SD: ±0.85)
Working as a student assistant has helped me to better understand clinical processes	3.45 (SD: ±0.86)
Working as a student assistant has helped me to better understand preoperative and postoperative measures	2.83 (SD: ±0.76)
Working as a student assistant has helped me to better understand certain orthopedic and trauma surgery clinical pictures	3.31 (SD: ±0.98)
Working as a student assistant motivated me to be scientifically active	2.29 (SD: ±1.13)

When asked to rank what influenced their further career the most, the majority of the participants stated that it was their experience in their final practical year (40.5%). This was followed in 16.7% of the participants who state that their experiences as student assistant in orthopedic surgery was most influential. The activity as student assistant in orthopedic surgery was ranked as the second and third most influential part of further career decisions by 28.6% of participants. When asked whether the participants would recommend other students to work as student assistant in orthopedic surgery, 78.6% stated yes.

## Discussion

Surgical specialties in Germany are already facing a shortage of residents [1]. It seems obvious that the groundwork to counteract this trend should be laid during the medical studies, especially when considering the fact that the interest in surgical residencies among medical students in Germany diminishes continuously as they progress through their studies [15]. This could be explained by the image of its physically demanding nature, long working hours, high stress levels, and the extended training period required, which can impact the work-life balance [18]. This underlines the need for sustainable measures to retain and potentially increase the number of medical students pursuing surgical residencies. In an evaluation by Kasch et al. the authors stated that personnel acquisition starts early in the medical field and that by improving the internship experience, students might be attracted towards a certain medical field and postgraduate specialist training [7]. As a possibility to early personnel acquisition, it has been suggested that student jobs can be a method of young talent recruitment [13]. This study examined how student roles as ward and operating room assistants in an orthopedic surgery department impacted medical students’ career choices and highlighted the benefits they gained from this position.

Firstly, the study showed that 50% of participants who had completed their medical studies selected a surgical specialty for residency, with most opting for orthopedic surgery. Given that previous research indicates that only 13% of medical students in Germany express interest in a surgical residency by the end of the ninth semester, this represents a significantly higher percentage [15]; however, this number must also be acknowledged with caution as most of the participants stated that they were interested in orthopedic surgery before they started the student job. There might have therefore been a certain selection bias.

Secondly, it was shown that apart from the practical year, the experiences gained during the student job had the strongest influence on participants’ future career choices. Student assistant roles had a moderately positive influence on participants’ career choices. While not a strong endorsement, this indicates that many participants found these roles to be helpful in shaping their career interests and guiding them toward a specific field of specialization. Nevertheless, most of the participants would recommend the student job to other students.

Furthermore, it was shown that engaging as a student assistant has provided the participants with substantial practical experience. The respondents stated that they have profited the most from the student job as it has helped them to learn practical skills on the ward, such as blood sampling, peripheral intravenous catheters, etc. Furthermore, it was shown that it has helped students to learn practical skills in the operating room, to better understand clinical processes and to learn skills at the level of clinical-practical communication and organization. The position proved less effective in fostering scientific activity and in developing the specialized skills required for comprehensive orthopedic and trauma-specific examinations.

The results of this study show that structured student assistant positions can be an effective tool to foster interest and practical skills in surgical specialties. To fully leverage the potential of such positions, several aspects should be considered in their design. A variety of tasks are crucial as combining activities in the operating room and on the ward provides students with comprehensive insights into daily clinical work and processes. Awareness of this effect as well as the shortcomings in current recruitment methods for young talent is crucial to improving strategies for attracting and retaining young doctors in the field [6]. Previously it has been shown that student’s exposure to surgery positively influences their interest in surgery [18]. After exposure students were more aware of what impact surgery has for patients and ensures that surgery enables a work-life balance [12]. Therefore, the student job may be a good option to introduce students to the surgical lifestyle and diminishing prejudgements that might exist for surgical residencies and thereby fostering stronger connections with the field. Zambare et al. have also shown that focused preclinical surgical exposure changes the student’s perceptions of surgery [19]. They have shown that surgical exposure can improve the perception of the length and difficulty of surgical training, gender inclusivity and patient-centered care, which in turn can increase their interest in surgical fields [19].

Moreover, mentorship programs could serve as a valuable addition, enabling experienced physicians to guide students and provide them with a deeper understanding of the specialty. Schmidt et al. have identified mentorship and experience in surgery as possible factors that can help attract medical students to peruse a surgical career [14]. Incorporating scientific activities, such as small research projects, could further help develop a broader range of skills. Furthermore, regular feedback on the students’ performance and development should be offered. This can help to support students’ learning progress.

To effectively position these programs they should be closely aligned with existing measures for recruiting young talent, such as the upcoming curricular reforms [2, 3]. For instance, integrating student assistant programs with enhanced curricular teaching or extracurricular workshops could create a more cohesive and supportive framework to attract students to surgical specialties. By linking these initiatives, institutions can maximize the impact of both structured practical experiences and broader educational reforms.

For broader implementation of such incentives, standardization is however necessary. Centralized guidelines, supported by professional societies, such as the German Society for Orthopedics and Trauma Surgery (DGOU), could establish minimum requirements and recommended contents. Another important step would be fostering interdisciplinary collaboration, where similar assistant programs could be introduced in other surgical and nonsurgical fields. Finally, regular evaluation of these programs is crucial. Using standardized surveys and feedback, the programs can be continuously improved and adapted to the needs of students.

There are several limitations to this study. Firstly, this was a monocentric study and the sample size was relatively small, so that the sample may not represent the broader population of medical students in Germany. In addition, interest in a surgical career could be biased as most participants stated that they had been interested in orthopedic surgery before working as a student assistant. Additionally, the study only focusses on assistantships in orthopedic surgery and no other surgical fields. While some of the knowledge acquired in this study can be translated, there might be differences that are specific to other surgical fields. In addition, the study only focuses on a single institution and may not account for variations in student experiences or recruitment practices across different medical schools in Germany. Therefore, multicentric studies also including medical students doing assistantships in different surgical fields are needed to verify and generalize the presented results. Additionally, the study relies on self-reported data, which can be subject to bias, such as recall bias or social desirability bias, where participants may overstate the influence of their student assistant experience on their career decisions.

## Conclusion

The findings suggest that structured student assistantships in orthopedic surgery departments may play a vital role in countering the decline in interest in surgical careers among medical students. It has been shown that these student jobs can foster practical technical skills in the operating room and on the ward, learning skills at the level of clinical practical communication and organization and better understand clinical processes. Offering student assistant jobs can therefore also be useful in terms of recruiting junior staff for our specialty.

## Supplementary Information

Questionnaire

## Data Availability

Data are available upon reasonable request.

## Abbreviations

DGOU: Deutsche Gesellschaft für Orthopädie und Unfallchirurgie (english: German society for orthopedics and trauma surgery)
SD: Standard deviation

## Declarations

For this article no studies with human participants or animals were performed by any of the authors. All studies mentioned were in accordance with the ethical standards indicated in each case.

## References

[1] Braun BJ (2019) Young surgery and the bottleneck of finding new blood. Innov Surg Sci 4(1):1–2. 10.1515/iss-2018-0025 31579794 10.1515/iss-2018-0025 PMC6754060

[2] Fröhlich S, Obertacke U, Rüsseler M, Walcher F, Seemann R (2023) Nationaler kompetenzbasierter Lernzielkatalog (NKLM) und neue Ärztliche Approbationsordnung ÄApprO 2025 – ein Wegweiser für O & U. Z Orthop Unfall 161(2):121–126. 10.1055/a-2017-1524 37015236 10.1055/a-2017-1524

[3] Ghanem M, Seemann R, Fröhlich S, Heyde C‑E, Roth A (2024) Die neue Ärztliche Approbationsordnung ÄApprO: aktueller Stand und zu erwartende Herausforderungen. Orthopädie 53(5):311–316. 10.1007/s00132-024-04495-6 38546842 10.1007/s00132-024-04495-6

[4] Histing T, Jung J, Wincheringer D, Ludwig J, Pizanis A, Pohlemann T, Menger MD (2013) Praktische, berufsrelevante Kurse für Studierende können nicht nur die Lehre verbessern, sondern steigern auch die Attraktivität des Faches Orthopädie/Unfallchirurgie. Z Orthop Unfall 151(4):389–393. 10.1055/s-0033-1350747 23963986 10.1055/s-0033-1350747

[5] Jähne J, Mittelstädt A, Götzky K (2017) Studentenforum und Kongressstipendien und ihre mögliche Bedeutung für die (chirurgische) Berufswahl. Chirurg 88(11):950–955. 10.1007/s00104-017-0520-2 28980035 10.1007/s00104-017-0520-2

[6] Kasch R, Wirkner J, Meder A, Abert E, Abert M, Schulz AP, Walcher F, Gümbel D, Obertacke U, Schwanitz P, Merk H, Froehlich S (2016) Wer bleibt nach einer Famulatur in Orthopädie und Unfallchirurgie dem Fachbereich treu? Ergebnisse einer bundesweiten Umfrage. Z Orthop Unfall 154(4):352–358. 10.1055/s-0042-104119 27294478 10.1055/s-0042-104119

[7] Kasch R, Abert E, Kolleck N, Ghanem M, Froehlich S, Hofer A, Schulz AP, Wassilew G, Herbstreit S (2021) Praktisches Jahr in Orthopädie und Unfallchirurgie – die kollegiale Staffelstabübergabe in die Weiterbildung? Z Orthop Unfall 159(6):624–630. 10.1055/a-1200-2544 32968989 10.1055/a-1200-2544

[8] Klepka KL, Siebenlist S, Kugler A, Reppenhagen S, Youssef Y (2024) Einfluss eines studentischen Curriculums auf den beruflichen und wissenschaftlichen Werdegang von Ärzt:innen. Arthroskopie 37(3):191–196. 10.1007/s00142-024-00679-6

[9] Kraus M, Böcker W, Youssef Y, Faber S (2024) Kontroversen in der Nachwuchsförderung in der Orthopädie und Unfallchirurgie. Orthopädie 53(5):317–323. 10.1007/s00132-024-04500-y 38634951 10.1007/s00132-024-04500-y

[10] Meder A, Lammerding-Köppel M, Zundel S, Stöckle U, Bahrs C, Gonser C (2016) Kann mit praxisorientierter curricularer Lehre das Interesse am Fach Orthopädie und Unfallchirurgie geweckt werden? Z Orthop Unfall 154(6):618–623. 10.1055/s-0042-111007 27612313 10.1055/s-0042-111007

[11] Merschin D, Mutschler M, Stange R, Kopschina C, Schüttrumpf JP, Doepfer AK, Achatz G, Niethard M, Hoffmann R, Kladny B, Perl M, Münzberg M (2016) Die Summer School der Deutschen Gesellschaft für Orthopädie und Unfallchirurgie – eine Erfolgsgeschichte. Z Orthop Unfall 154(5):499–503. 10.1055/s-0042-106477 27249045 10.1055/s-0042-106477

[12] Peel JK, Schlachta CM, Alkhamesi NA (2018) A systematic review of the factors affecting choice of surgery as a career. Can J Surg 61(1):58–67. 10.1503/cjs.008217 29368678 10.1503/cjs.008217 PMC5785290

[13] Samland M, Youssef Y (2021) Nachwuchsgewinnung neu gedacht. Z Orthop Unfall 159(2):137–138. 10.1055/a-1238-8753 33770819 10.1055/a-1238-8753

[14] Schmidt LE, Cooper CA, Guo WA (2016) Factors influencing US medical students’ decision to pursue surgery. J Surg Res 203(1):64–74. 10.1016/j.jss.2016.03.054 27338536 10.1016/j.jss.2016.03.054

[15] Schneider KN, Masthoff M, Gosheger G, Schopow N, Theil JC, Marschall B, Zehrfeld J (2020) Generation Y in der Chirurgie – der Konkurrenzkampf um Talente in Zeiten des Nachwuchsmangels. Chirurg 91(11):955–961. 10.1007/s00104-020-01138-2 32060578 10.1007/s00104-020-01138-2 PMC7581597

[16] Schüttrumpf JP, Münzberg M (2013) Pilotprojekt zur Nachwuchsgewinnung. Neues Wahlfach an der Universitätsmedizin Göttingen. Z Orthop Unfall 151(3):217–218. 10.1055/s-0033-1349247 23775498 10.1055/s-0033-1349247

[17] Thiele K, Matziolis D, Perka C (2010) Nachwuchsmangel in der Unfallchirurgie und Orthopädie. Ein Lösungsansatz. Unfallchirurg 113(12):1053–1056. 10.1007/s00113-010-1861-1 20842330 10.1007/s00113-010-1861-1

[18] Yudien MA, Brooks AD, Aarons CB (2024) Medical student perceptions of academic surgery: rose-colored glasses or jaded prism?p. J Surg Educ 81(3):373–381. 10.1016/j.jsurg.2023.11.020 38177035 10.1016/j.jsurg.2023.11.020

[19] Zambare WV, Dechert TA, Sanchez SE, Brahmbhatt TS (2021) Changes in medical student perceptions of surgery are sustainable through focused preclinical surgical exposure. J Surg Educ 78(5):1583–1592. 10.1016/j.jsurg.2021.02.008 33771474 10.1016/j.jsurg.2021.02.008

