# Supplementary material for: Student assistantships in orthopedic surgery: A strategy to counteract the declining interest in surgical careers
Source: Orthopadie (Heidelb). 2025 Mar 26;54(7):543–9. doi: 10.1007/s00132-025-04645-4 (PMC12269039; doi:10.1007/s00132-025-04645-4)
Supplement: Supplementary file 1 — Questionnaire [file 132_2025_4645_MOESM1_ESM.docx]

**Demographic Data**

1. **Gender:**
   o Male
   o Female
   o Diverse
2. **Age (in years):**
3. **Current Level of Education:**
   - 5th semester
   - 6th semester
   - 7th semester
   - 8th semester
   - 9th semester
   - 10th semester
   - Final Year (PJ)
   - 1st year of specialist training
   - 2nd year of specialist training
   - 3rd year of specialist training
   - 4th year of specialist training
   - 5th year of specialist training
   - 6th year of specialist training
   - More than 6 years of specialist training
   - Completed specialist training
     If yes, which specialty: ________________
   - Specialist training currently paused (e.g., research stay, parental leave, etc.)
   - Discontinued specialist training
4. **If you have already started a specialist training program: In which medical field are you currently working?**
   - Orthopedics and Trauma Surgery
   - Another surgical specialty (e.g., General Surgery, Vascular Surgery, etc.)
     If yes, which: ________________
   - A non-surgical specialty (e.g., Internal Medicine, General Medicine, etc.)
     If yes, which: ________________
   - No clinical activity (e.g., working in the industry)
   - Other
     If yes, please specify: ________________
5. **Do you intend to complete specialist training in the above-mentioned field?**
   - Yes
   - No, instead: ________________

**Student Assistant Activity**

1. **In which semester did you start working as a Student Assistant in the Department of Orthopedics and Trauma Surgery at the university clinic Leipzig?**
   - 5th semester
   - 6th semester
   - 7th semester
   - 8th semester
   - 9th semester
   - 10th semester
   - Final Year of internship (Praktisches Jahr – PJ)
2. **How long have you worked as a Student Assistant in the Department of Orthopedics and Trauma Surgery at the university clinic Leipzig? (If still working, duration until now)**
   - 6 months
   - 12 months
   - 1.5 years
   - 2 years
   - 2.5 years
   - 3 years
   - 3.5 years
   - 4 years
   - More than 4 years
3. **Were you interested in Orthopedics and Trauma Surgery before working as a Student Assistant?**
   - Yes
   - No
4. **What type of hospital or healthcare setting do you currently work in or plan to work in?**
   - University Hospital
   - Tertiary/Secondary Care Center
   - Primary Care Hospital
   - Specialized Clinic
   - Outpatient Practice
   - Other
5. **To what extent did your work as a Student Assistant in the Department of Orthopedics and Trauma Surgery at the university clinic Leipzig influence this decision?**

- Not at all
- Rather not
- Neutral
- Rather yes
- Yes, very much
- I don’t know

1. **Thinking about your career so far, which academic programs or mandatory components of your studies have had the most positive impact on your career?**
   [Rank from 1 to 5 (1 = strongest influence, 5 = weakest influence)]

- University courses (lectures, examination courses, etc.)
- Student Assistant job in the OUP
- Student Assistant job outside the OUP
- Clinical internship (Famulatur)
- Final Year of internship (Praktisches Jahr – PJ)
- Offers from medical societies

**Impact of the Student Assistant Activity**

1. **Statements regarding the impact of the Student Assistant activity on career and training:**

Likert scale from 1–5 (1 = Strongly disagree; 2 = Disagree; 3 = Neither agree nor disagree; 4 = Agree; 5 = Strongly agree)

- Working as a Student Assistant influenced my choice of current or desired specialty.
- Working as a Student Assistant helped me learn practical skills in the hospital ward (e.g., blood draws, IV cannulation, blood culture collection, etc.).
- Working as a Student Assistant helped me build and expand my professional network.
- Working as a Student Assistant helped me develop practical skills in the operating room.
- Working as a Student Assistant helped me conduct orthopedic/traumatological clinical examinations.
- Working as a Student Assistant helped me improve my clinical-practical communication and organizational skills.
- Working as a Student Assistant helped me understand clinical processes better.
- Working as a Student Assistant helped me understand pre- and post-operative measures better.
- Working as a Student Assistant helped me better understand specific orthopedic-trauma conditions.
- Working as a Student Assistant motivated me to pursue scientific research.

1. **Would you recommend working as a Student Assistant in a surgical department to students?**

- Yes
- No
- Not sure
